# Importance of Flow Metrics on Modeling Macroinvertebrate Community in Dammed Rivers: An Approach With Optimized Gradient Boosting

**DOI:** 10.1002/ece3.72411

**Published:** 2025-10-28

**Authors:** Kei Nukazawa, Ryo Tanaka, Haruki Mineda

**Affiliations:** ^1^ Department of Civil and Environmental Engineering, Faculty of Engineering University of Miyazaki Miyazaki Japan; ^2^ West Japan Engineering Consultants, Inc. Fukuoka Japan

**Keywords:** ecological niche modeling, hydrological simulation, indicators of hydrologic alteration, LightGBM, random forest algorithm, XGBoost

## Abstract

Habitat models that can predict the habitat suitability for riverine organisms and their distribution along environmental gradients are helpful in watershed environmental management. However, the impacts of dams on riverine communities and their habitats have not yet been considered in such models, although dams have considerably altered important riverine habitats. In this study, we aim to develop catchment‐scale habitat models of macroinvertebrate communities with predictor variables characterizing the impacts of dams. We studied the Omaru River catchment in southwest Japan, where the river flow has been altered considerably because of multiple hydropower dams. Multiple machine learning techniques, such as XGBoost and LightGBM were used to model the habitat distributions of 170 macroinvertebrate taxa observed throughout the river catchment. We used predictor variables of dam impacts derived based on geographical information system data (hereafter, dam metrics) and physically simulated flow data using a hydrological model. Among the modeling techniques, gradient boosting algorithms (XGBoost and LightGBM) with optimized tree number parameters exhibited the highest mean accuracy among the analyzed taxa, followed by the random forest algorithm. The accuracy of the habitat models for the macroinvertebrate community and habitat groups considerably improved with the integration of dam metrics and flow predictors. Of the habit groups considered, clingers showed a keen response to low‐flow metrics, presumably owing to flow alteration caused by the studied dams, as the downstream sections of these dams received only residual flow. Our findings indicate that (1) variables of dam impacts greatly improve the predictive capability of macroinvertebrates and (2) gradient boosting machines with optimized parameters are favorable for habitat modeling of the biotic community. Our models are helpful when river practitioners implement conservation measures as they better understand the environmental consequences of their flood protection designs.

## Graphical abstract

This study developed catchment‐scale habitat models of macroinvertebrate community with predictor variables that characterize dam impacts based on either simple geographical information system data or physically simulated flow data using a distributed hydrological model. Among the modeling techniques, XGBoost and LightGBM with optimized tree number parameters showed the highest mean accuracies among the taxa, followed by random forest. The accuracy of each algorithm greatly improved with the dam metrics and flow alteration metrics in many macroinvertebrates.

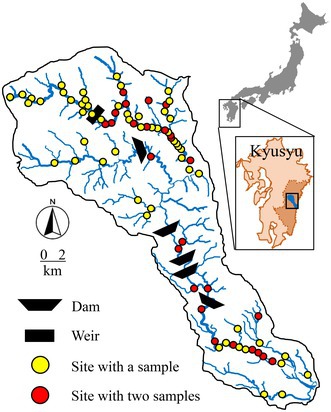

## 1 Introduction

Habitat models estimate habitat suitability or the presence/absence of species at locations where biological surveys have not been conducted. These models analyze the association between the spatial distribution of species and environmental attributes (e.g., temperature) based on statistical approaches (Elith and Leathwick 2009). In the past, researchers have developed riverine habitat models targeting broad to localized spatial scales (Bálint et al. 2011; Huang and Frimpong 2016; Manel et al. 1999), whereas there are only a few cases that target relatively small scales (e.g., small to medium catchments). The primary reason for this bias is the lack of extensive data for statistical modeling, such as limited biological data. For example, the Ministry of Land, Infrastructure, Transport and Tourism (MLIT), Japan, provides national census data for riverine organisms, including fish, benthos, and plants (the River Environmental Database); however, surveillance is performed on only a few sections of limited rivers important for flood protection. Therefore, to develop well‐trained habitat models along broader environmental gradients at a catchment scale, considerable effort is needed to collect extensive spatial data from upstream to downstream while considering seasonality.

The abovementioned problem of scarce data can be addressed by targeting macroinvertebrates because collecting samples over a limited period requires relatively low effort. Riverine macroinvertebrates have been widely used as the biological indicators of specific environmental impacts (C. Wang et al. 2023), suggesting that they have an advantage in structuring habitat models to assess these impacts. Although habitat models targeting macroinvertebrates have been developed in the past (Cha et al. 2021; Domisch et al. 2013; Gogina and Zettler 2010), habitat models that can be applied to other catchment regions (i.e., “transferable”) (Hao et al. 2020) are yet to be accomplished. This lack of transferable habitat models is partially due to the insufficient consideration of environmental factors, such as hydrological variation and artificial impacts, which are relatively more ecologically relevant to macroinvertebrate habitats (Guisan and Zimmermann 2000; Jarnevich et al. 2015). Although earlier studies have applied hydrological predictors (Kuemmerlen et al. 2015; Nukazawa et al. 2023, 2018), the development of transferable models remains challenging owing to the limited data availability.

Other significant abiotic factors that preclude the transfer of riverine habitat models include a variety of anthropogenic impacts, such as water pollution and riverbed degradation caused by land‐use alteration and damming (Dudgeon et al. 2006; Jelks et al. 2008). Climate change is an anthropogenic effect that has frequently been integrated into habitat models (Kishore et al. 2024), although its efficacy remains uncertain when modeling aquatic habitats using climate variables (Nukazawa et al. 2023). In contrast to studies using climate attributes, studies that consider the environmental impacts of dams are limited. The effects of dams on streams are evident as changes in stream environments through various pathways and the responses of organisms to these changes. Several studies have demonstrated the consequences of such alterations. For example, changes have been reported in macroinvertebrate communities due to reduced algal production owing to prolonged dam‐induced turbidity in stream water (Armitage 1977; Jones et al. 2019) and in both macroinvertebrate and fish communities due to alterations in streamflow (Nukazawa, Shirasaka, et al. 2020). However, it requires considerable effort to fully understand the complex effects of dams on riverine communities; therefore, it is fruitful to examine the predictive ability of simple and accessible dam impact metrics.

In this study, we utilized previously developed simple dam metrics (Cooper et al. 2017) and the flow indicators of hydrologic alteration (IHA) (Richter et al. 1996) to address the issue of acquiring data representing the complex impacts of dams. Dam metrics can be derived from fundamental geographic information system (GIS) data and represent the combined impact of a dam. For instance, such metrics have been used successfully to quantify the decline in stream connectivity caused by damming (Cote et al. 2009), the extent of flow regulation (Lehner et al. 2011), and the number of dams per river length in a catchment (hereafter referred to as dam density) (Van Looy et al. 2014). Flow regime metrics, including IHA, have been widely used (Carlisle et al. 2017) to assess the impact of flow regulation on macroinvertebrates (Nukazawa, Shirasaka, et al. 2020; Schneider and Petrin 2017) and that of tributary confluences on fish population dynamics in the Missouri River (Pracheil et al. 2009). In the recent past, dam metrics have been applied in habitat modeling studies. In a previous study, Daniel et al. (2018) developed habitat models of freshwater mussels using dam metrics and reported that upstream dam density adversely impacts the species. Another report warned that dam density decreases the probability of fish occurrence (Mollenhauer et al. 2021). The authors integrated flow regime metrics and reported that the declining habitat is ameliorated by increased downstream flow. Furthermore, a recent study successfully predicted habitat gains due to dam removal and the construction of fish passes based on a similar habitat modeling framework (Cooper et al. 2021). However, to our knowledge, dam metrics have not yet been applied to the macroinvertebrate community, and thus, the predictive ability of such metrics is not understood. Capturing an ecological niche led by complex spatial distribution patterns of community and background environmental heterogeneity is a major challenge, and recent advances suggest sophisticated machine learning approaches, such as deep learning, and the variants of existing algorithms, e.g., light gradient boosting machine (LightGBM) (Ke et al. 2017). Applying these techniques to models of macroinvertebrate community allows us to understand the usefulness of newly proposed predictors as the methods offer greater options for optimizing individual models of taxa.

In this study, we developed the habitat models of macroinvertebrate community using dam metrics and flow metrics for the Omaru River catchment in southwest Japan. We hypothesized that the contributions of new predictors would vary among macroinvertebrate taxa owing to a variety of traits (e.g., habit) (Poff et al. 2006). Therefore, we examined the contributions of predictors to the accuracy of habitat models in the habitat groups of macroinvertebrates. We adopted relatively new machine learning techniques, LightGBM and XGB, and optimized the key parameters. The findings of this study demonstrate the usefulness of such optimization in modeling multiple taxa in a specific community.

## 2 Materials and Methods

**FIGURE 1:**
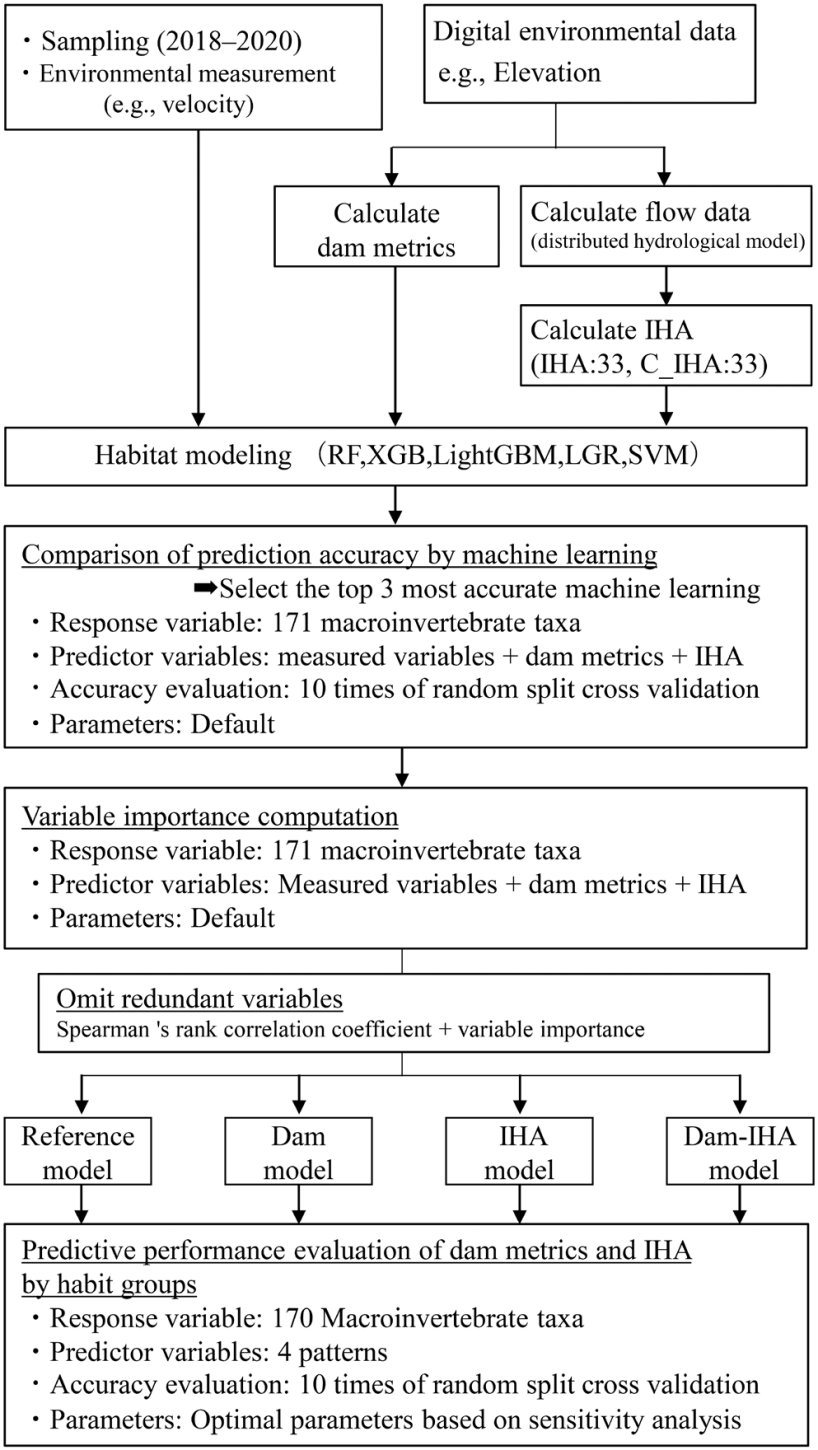
Flowchart depicting the study design.

Herein, we developed the habitat suitability or species distribution models of macroinvertebrate taxa based on presence/absence data sampled throughout a catchment and various environmental variables observed and acquired from geographical information systems and hydrological simulations. Detailed data used for modeling are available in a repository as Tables S1 and S2 (https://doi.org/10.34481/0002001366). Using these models, we explored the predictive ability of dam‐impact variables, such as dam density in upstream watersheds and flow regimes. Figure 1 summarizes the study design.

### 2.1 Study Area

**FIGURE 2:**
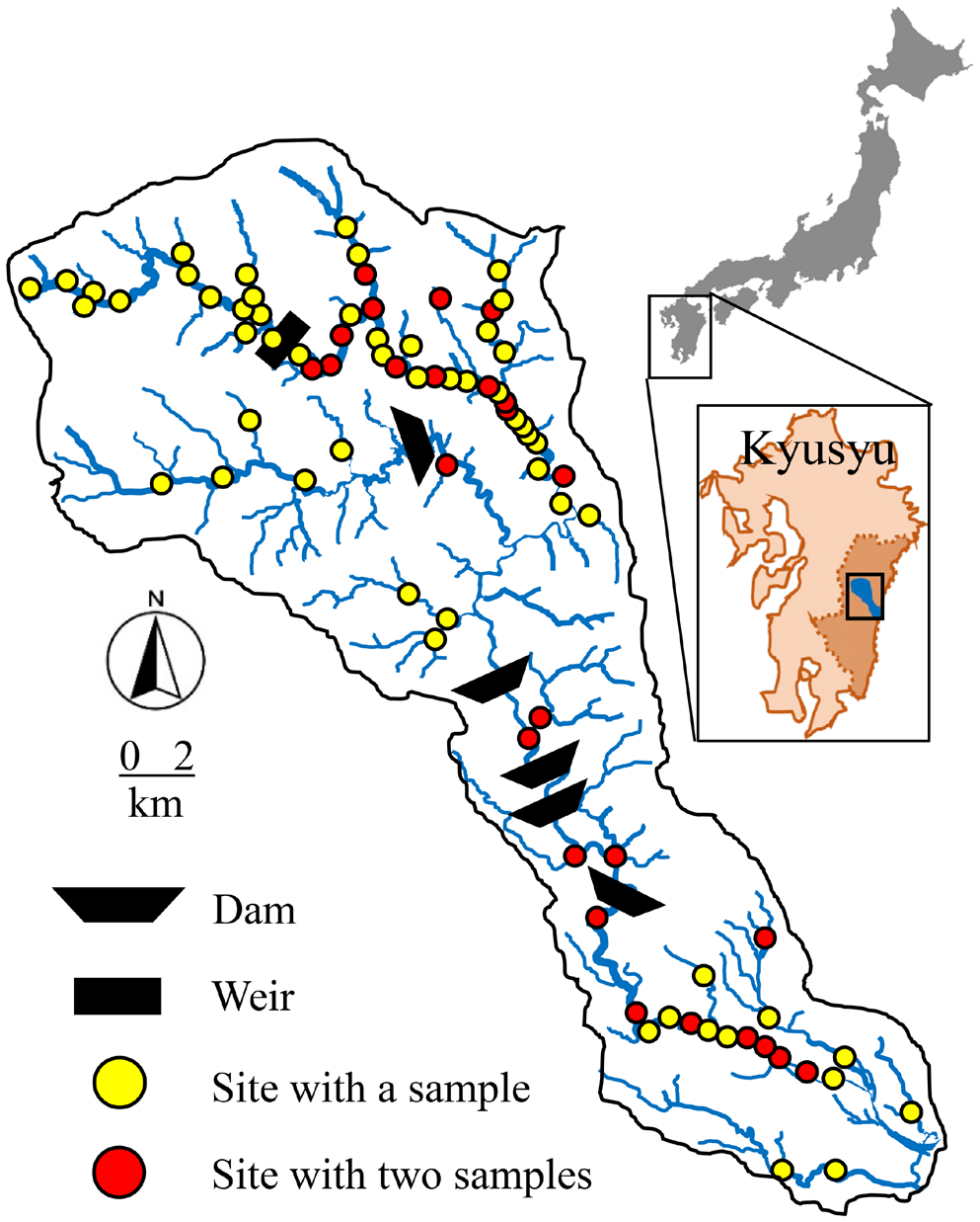
Spatial allocations of the sampling sites, dams, and weir in the Omaru River catchment.

We studied the Omaru River catchment in southwestern Japan (474 km^2^) (Figure 2). The main stem stretches 75 km from the steep upland topology to the lower lands with a moderate slope (1/600–1/1000). The mean annual temperature and annual precipitation are approximately 17.6°C and 2300 mm at the Takanabe meteorological station in the lowland area (http://www.qsr.mlit.go.jp/miyazaki/kasen/omaru/gaiyou/omaru_saigai.html). The catchment population is approximately 31,000, which is mostly concentrated in the downstream area. The land use/land cover is dominated by forests (ca. 87%), followed by agricultural land (10%) and urbanized regions (3%). Five dams in the mainstem and tributary (the Do River) were used for flood control, hydropower generation, and irrigation (Mineda et al. 2023).

### 2.2 Macroinvertebrate Sampling

Macroinvertebrate sampling was performed at 60, 22, and 25 sites in November 2018, 2019, and 2020, respectively. We fixed the sampling season to exclude the effects of the changes in community composition due to life‐cycle events, such as emergence during sampling durations, and to minimize the impacts of disturbances caused by flooding that typically occur from summer to early autumn in this region. Notably, during and before the sampling period, a negligible amount of rainfall was observed in 2018 (1.5 mm/day at the upland Mikado meteorological station) and 2019 (4.5 mm/day at the lowland Takanabe meteorological station), and no rainfall was observed in 2020. We selected 82 sampling sites from the uppermost to downstream reaches, classified into 62 riffle sites (i.e., riffle‐dominated sites), 14 pool sites, and 6 riffle–pool sites (i.e., sites with an even distribution of riffles and pools) (Figure 2). Of the 82 sites, we sampled twice at 25 sites, including 19 riffle sites and 6 pool sites, with broad environmental gradients in 2020 (*n* = 107).

We performed a conventional quantitative sampling using a quadrat Surber sampler (25 cm × 25 cm, *φ* = 500 μm) for macroinvertebrate collection at riffles and pools. At the riffle sites, the sampler was set at four arbitrary points with a flow velocity of > ca. 0.2 m/s, and macroinvertebrates were sampled following the protocol specified in the River Environmental Database of MLIT, Japan. Briefly, gravel of size larger than 20 mm and cobbles were washed off, and the riverbed was disrupted for 20 s while invertebrates were collected using an attached net. At the pool sites, the sampler was set at four arbitrary points, and macroinvertebrates were sampled by disrupting the riverbed by foot for a minute due to a greater water depth (> ca. 0.35 m) than that at the riffle sites. The sampled macroinvertebrates were identified using a microscope to the lowest feasible taxonomic level (usually the species or genus level). Subsequently, the taxa were classified into habit groups following the published literature (Table S3) (Takemon 2005), which is an optimized classification of Japanese macroinvertebrate taxa based on a previous study (Merritt and Cummins 1996).

### 2.3 Environmental Measurements

Environmental attributes were measured on the same dates as those for macroinvertebrate sampling and listed in Table S4. Note that we could not measure partial parameters (e.g., velocity; see Section 2.6.2) at the two sites due to a technical problem. We measured the current velocity using a flowmeter (VR‐30J; KENEK, Tokyo, Japan) and water depth at 4–5 points at each site and then derived the average values. The water temperature was measured at each site. We sampled 250 mL of flowing water and measured the pH and electrical conductivity (EC) using a pH meter and conductivity meter, respectively (Laqua Horiba, Kyoto, Japan).

In addition to these measures, we visually determined the six site categories: riffles/pools, riverbank vegetation, canopy openness, levees, and bedrock. The riffle/pool sites consisted of three classes, i.e., “riffle sites,” “pool sites,” and “riffle–pool sites.” The riverbank vegetation consisted of three classes, i.e., “no vegetation,” “sparse vegetation,” and “dense vegetation.” The canopy openness consisted of three classes, i.e., “no canopy,” “sunlight somewhat available,” and “sunlight unavailable.” The levee consisted of three classes, i.e., “no levee,” “more than half of the site section is covered by levee at either bank,” and “more than half of the site section is covered by levees at both banks.” The bedrock consisted of two classes, i.e., “no bedrock” and “covered by bedrock.”

We measured the substrate material size at a riverbank sandbar as a proxy for the riverbed material size because of the considerable effort and cost associated with collecting riverbed data in our sampling design. For the measurements, we used a 1 m × 1 m quadrat with 10 cm intervals. The major and minor axes of the materials were measured under each cross‐section, and the mean material size was derived.

### 2.4 Geographic Information System Data

#### 2.4.1 Fundamental Geographical Attributes

Land use was determined using an approximately 10‐m resolution dataset of the global digital surface model collected by the Advanced Land Observing Satellite (ALOS) (https://www.eorc.jaxa.jp/ALOS/en/index_e.htm). A hydrologically corrected digital elevation model (DEM) with a spatial resolution of 1 s (approximately 30 m) (Yamazaki et al. 2020) was used to determine the catchment area and slope at a 250 m resolution throughout the Omaru River catchment. Here, we derived the two types of slopes based on the differences between the sampling site and the grid cell with the highest or lowest altitude among the surrounding eight meshes of the DEM. The digital environmental data used as predictor variables for the habitat models are listed in Table S4.

#### 2.4.2 Dam Metrics

Sixteen dam metrics (Table S5), developed in a previous study (Cooper et al. 2017), were applied to the Omaru River catchment. The metrics were derived using data on the number and location of dams, the river network, reservoir storage, and catchment area, and were classified into four groups. The first group included metrics for the sizes of river sections fragmented by dams (e.g., total segment length fragmented by dams). The second group included metrics for the number and density of dams (e.g., the number of upstream dams). The third group included metrics for the distance to the dam (e.g., distance to the nearest dam upstream of the main stem). The fourth group included metrics for reservoir storage (e.g., total reservoir volume upstream per total length of upstream rivers).

However, of these 16 metrics, 7 metrics could not be computed at all sites (e.g., computed only for the main stem) because of their target locations. Therefore, these metrics were excluded from the habitat modeling procedures to keep the sample size required for robust model development. The river length was measured from each site to the upstream and downstream dams using the digital national land information for the river (nlftp.mlit.go.jp/ksj/) on QGIS ver. 3.1.2. We acquired reservoir storage data from the webpages of the Kyushu Electric Power Co. Inc. (http://www.kyuden.co.jp/company_outline_branch_miyazaki_initiative_power_01.html) and Miyazaki Prefecture (https://www.pref.miyazaki.lg.jp/kasen/shakaikiban/kasen/dam.html).

### 2.5 Flow Metrics Derived Based on Hydrological Simulations

As predictor variables, we used daily flow data at each sampling date, the IHA, and the ratio of change in IHA. The IHA is an assemblage of 33 stream flow indices that assess specific flow alterations and is classified into five categories corresponding to the impacts on riverine ecosystems: e.g., seasonal flow variation patterns (i.e., median flow in each month), extreme flow (e.g., drought and flooding), and magnitude and duration (e.g., maxima/minima of 1–90 day moving averages; 1–90‐Max, 1–90‐Min) (Richter et al. 1996). The IHAs were computed for every calendar year corresponding to the sampling years (i.e., 2018, 2019, and 2020) (iha, R ver. 3.6.3) based on daily flow data simulated using a distributed hydrological model (Mineda et al. 2023). The hydrological model includes flow manipulation by dams using outflow data from dam outlets and hydropower plants and could reproduce the streamflow time series at an arbitrary point in the watershed. The model was verified to be sufficiently accurate (Nash–Sutcliffe efficiency coefficient > 0.7) throughout the Omaru catchment including watercourses downstream of the dams. We considered the change ratio of each IHA with/without dams derived from hydrological analyses either using the initial conditions for dam‐induced hydrological alterations (e.g., dam outflow) or not. The change ratios were derived using the following equations:

(i) If the unit of IHA is m^3^/s, number, and days,

(1) $$R_{1}=\frac{{\mathrm{IHA}}_{1}-{\mathrm{IHA}}_{0}}{{\mathrm{IHA}}_{0}}\times 100$$

(ii) If the unit of IHA is day,

(2) $$R_{2}={\mathrm{IHA}}_{1}-{\mathrm{IHA}}_{0}$$

where *R*_1_ is the change ratio (%), *R*_2_ is the extent of change (day), IHA_0_ is the IHA derived from the daily flow data computed without dam data, and IHA_1_ is IHA derived from the daily flow data computed with dam data. Hereafter, *R*_1_ and *R*_2_ will be denoted as C_IHA (change ratio/extent of IHA). Partial C_IHA with missing values was excluded from the subsequent modeling process. Detailed information on these metrics (Mineda et al. 2023) is provided in Table S6.

### 2.6 Habitat Modeling

#### 2.6.1 Machine Learning Techniques

We applied the following five regression and machine learning techniques to model the distribution of macroinvertebrates: random forest (RF), XGBoost (XGB), LightGBM, logistic regression (LGR), and support vector machine (SVM). The RF algorithm (randomForest, R ver. 4.1.2) (Breiman 2001) is an ensemble learning technique with bootstrap aggregation, which develops numerous decision trees through bootstrapping and predictions based on majority voting (classification) or averaging (regression). We adopted RF as it outperforms other machine learning classification techniques with several response variables in many disciplines (Zhang et al. 2017), is suitable for modeling with many predictor variables (Feld et al. 2016), provides variable importance metrics, and is robust against overfitting.

The XGB algorithm (xgboost, Python ver. 3.10.7) (Chen and Guestrin 2016) is a variant of the gradient boosting machine (GBM), which is an ensemble machine learning model with a boosting technique. Boosting is an approach for sequentially developing decision trees while considering the loss functions of a previous decision tree to improve model performance. Although the fundamental GBM is generally time‐consuming because of its sequential modeling approach, XGB uses a weighted quantile sketch algorithm to address this issue. In addition to RF, XGB also outperforms other machine learning classification techniques with a variety of response variables in many disciplines (Zhang et al. 2017) and is suitable for modeling with many predictor variables (Maloney et al. 2012).

The LightGBM algorithm (lightgbm, Python ver. 3.10.7) (Ke et al. 2017) is based on GBM and differs from XGB in the process of generating decision trees. The XGB algorithm applies a “level‐wise” procedure that splits all branches at a level and then moves to the next level, whereas LightGBM applies a “leaf‐wise” procedure that splits nodes to minimize a loss, enabling efficient learning.

The LGR model (LogisticRegression, Python ver. 3.10.7) (Cox 1958), also known as a generalized linear model, is a binary classification technique based on a sigmoid function. The SVM (SVC, Python ver. 3.10.7) (Cortes and Vapnik 1995) is a classification method that maximizes the margins between classes. Both algorithms were conventionally used for binary classification problems before the proliferation of decision tree‐based models. Therefore, we adopted these algorithms to compare with the decision tree algorithms applied in this study.

#### 2.6.2 Interpolating Predictor Variables

Before modeling the macroinvertebrate habitats, missing data in the partial predictor variables at the two sites (*n* = 2; 1.9%) were interpolated. Interpolation was performed for six environmental variables, i.e., flow velocity, water depth, pH, EC, water temperature, and substrate materials using RF because it has performed well in many fields. Predictor variables were selected based on those used for benthic macroinvertebrates due to limited prior information on predictive factors. As the water temperature was measured at different times of the day, we added the sampling time of the day as a predictor of water temperature. In total, the interpolation was performed using 19 environmental variables. The accuracy of the models was evaluated in terms of Pearson's correlation coefficient between the observed and predicted values based on the 10 rounds of random split cross‐validation (80% training and 20% validation) (Figure S1). In general, the flow velocity, water depth, and substrate materials were relatively well modeled, whereas the accuracy of the other variables was limited.

#### 2.6.3 Comparison of Accuracies Among the Modeling Algorithms

First, we built presence/absence habitat models for 171 macroinvertebrate taxa (https://doi.org/10.34481/0002001366) recorded as present in more than three samples with all predictor variables using the five modeling algorithms. Subsequently, the mean accuracies of the 171 taxa were compared among the five regression/machine‐learning algorithms. To do so, we derived the area under the curve (AUC) of the receiver operating characteristic (ROCR, R ver. 4.1.2) for the 10 rounds of random split cross‐validations (80% training and 20% validation) for each taxon. The algorithms with greater AUC values than 0.7, which is a criterion of fair prediction accuracy (Swets 2015), were used for the downstream analyses.

#### 2.6.4 Contributions of the Dam Metrics and IHA and Parameter Setting for Machine Learning

The mean variable importance measures were calculated for each predictor variable based on RF and LightGBM (RF: MeanDecreaseAccuracy, LightGBM: split) among the 171 models to evaluate the contributions of dam metrics and IHA to the prediction of macroinvertebrate habitats. XGBoost was not applied here because it has been reported to generate biased feature importance (Takefuji 2025). Subsequently, we built the following four models: (1) a model that excludes the dam index/IHA and half of the other variables with low importance (hereafter, the reference model), (2) a model that adds the dam metrics to the reference model (hereafter, the dam model), (3) a model that adds the IHA to the reference model (hereafter, the IHA model), and (4) a model that adds both dam metrics and IHA to the reference model (hereafter, the dam–IHA model). These four models were compared for their prediction accuracies. Selecting non‐redundant variables out of the larger set of variables such as IHA is key to adequately understanding the impacts of such variables on macroinvertebrates (Olden and Poff 2003; Kakouei et al. 2018). Therefore, among the variables with a higher Spearman's rank correlation than the criterion (> 0.7), those with low importance in RF and LightGBM were excluded to decrease model redundancy. The correlation matrix among the metrics is shown in Figure S2. As the number of IHA‐related metrics was large (> 60), the two most important variables for each IHA group were retained. In brief, considering the cross‐correlation and variable importance, five variables, such as elevation, were selected in the reference model; four additional variables, such as upstream network dam density per unit network catchment area (UNDC), were selected in the dam model; nine additional variables, such as the median discharge of September, were selected in the IHA model; and all variables in the dam and IHA models were selected in the dam–IHA model (Tables S4–S6).

Sensitivity analyses were performed to identify the optimal hyperparameters for XGB and LightGBM to attain better modeling accuracy for the entire community of macroinvertebrates. Note that we did not include the RF for sensitivity analyses because hyperparameters in this algorithm were found to be low in our preliminary analyses. As a result, we identified the two major hyperparameters: the number of features and the number of trees as 2 and 500, respectively. In both algorithms, because of the need for substantial effort and high computing cost for optimizing a vast number of parameters (> 100), we first focused on the parameters that were considered in previous studies (Bentéjac et al. 2021; Qiu et al. 2021) (Table S7). Each parameter was varied individually, and the two most sensitive parameters were chosen for the subsequent in‐depth sensitivity analyses. In addition to the abovementioned two sets of parameters, for the sensitivity analyses, we used a hyperparameter “early_stopping_round” (hereafter, ESR) that stops the boosting process when it does not improve the performance, as ESR improved the accuracy of the community model in our preliminary analyses. A hyperparameter “num_boost_round” for the number of trees was assigned a large value (10,000) in this process such that ESR works over a wide range of tree numbers. The accuracies were compared by changing the combination of the three parameters, and the combination with the highest accuracy was identified as the final set of parameters. In the sensitivity analyses, we used the predictor variables for the dam–IHA model.

Notably, XGB tended to show a high prediction accuracy, along with a relatively small subsample rate (SR) and the proportion of randomly extracted samples (Figure S3), suggesting that a small SR can avoid overfitting; thus, better accuracy was obtained with the test data. The accuracy decreased when the SR changed from 0.5 to 0.15 (only 15% of the data used) because the data were insufficient for learning, and the accuracy decreased, as indicated in a systematic review of environmental applications (Xu and Liang 2021). The accuracy greatly improved as the ESR increased, regardless of the variations in the SR and learning rate (LR). In attempts at LightGBM, the accuracy increased with a relatively small feature fraction (FF) and the ratio of randomly extracted features (i.e., predictors) (Figure S4). This implies that variables with high predictive ability are selectively used under a high FF, resulting in a large model bias and low accuracy. High LR and ESR in LightGBM generally increased the accuracy, as observed in XGB.

The positive contribution of the relatively high LR observed in both XGB and LightGBM was strongly associated with the number of trees and ESR. In general, when the LR was high, a relatively small number of trees exhibited a higher predictive performance than when the LR was low. When ESR = 0, the models were developed with a designated number of trees (i.e., 10,000), which is a relatively large number; thus, the accuracy decreased with a high LR. However, when ESR was activated (ESR = 10–1000), learning in many models stopped in the early learning stages (the number of trees was < 1000). Therefore, the mean accuracy increased with a high LR implemented with ESR through optimizing the important hyperparameters (number of trees) for each taxon. Based on the abovementioned considerations, the hyperparameters used to evaluate the contributions of the dam metrics and IHA to the distribution of macroinvertebrates were identified (Table S7).

The response variable for the sensitivity and contribution analyses of the dam metrics and IHA was the presence or absence of 170 macroinvertebrate taxa. A taxon that could not be classified as a habit (Chironomidae sp.) was excluded during model development. The prediction accuracy among the 170 taxa was evaluated based on the AUC for the 10 rounds of random split cross‐validation (80% training and 20% validation).

## 3 Results

### 3.1 Comparison of Prediction Accuracy Among Modeling Algorithms

**FIGURE 3:**
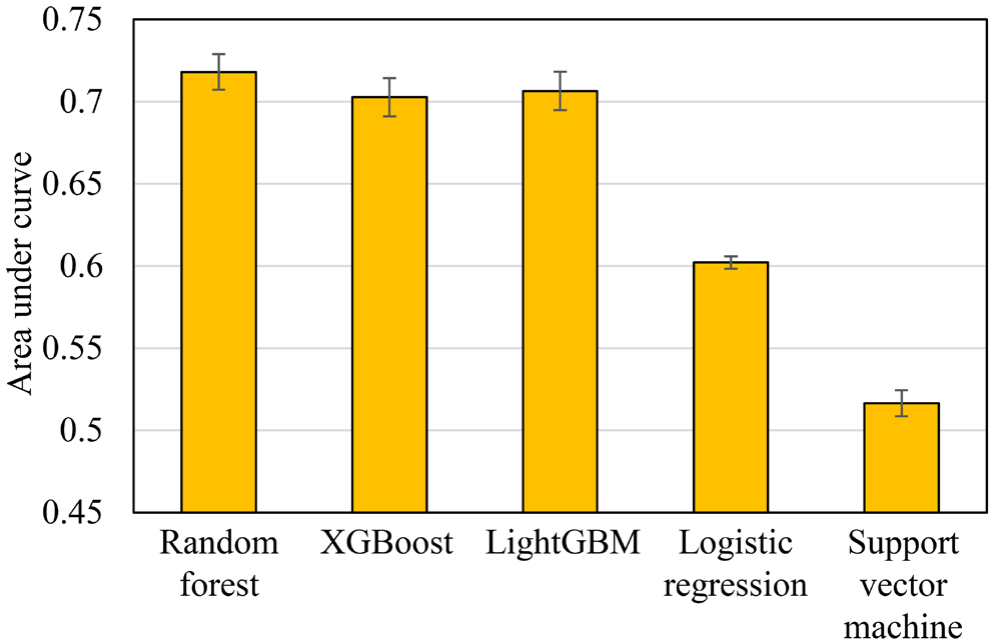
Mean and standard error of area under curve (AUC) in the models of 171 macroinvertebrate taxa integrating all predictors for each algorithm.

The mean AUC values among the 171 macroinvertebrate taxa for each modeling algorithm with all predictor variables but without any parameter tuning showed that decision‐tree‐based ensemble learning techniques (i.e., RF, XGB, and LightGBM) outperformed the other algorithms with AUC values greater than 0.7, which is a criterion of fair prediction accuracy (Swets 2015) (Figure 3). The lower accuracies of LGR and SVM than those of RF, XGB, and LightGBM were specifically characterized by taxa occurring in a few samples, suggesting the limitations of these algorithms in delineating classification boundaries. Therefore, the RF, XGB, and LightGBM algorithms were used for downstream analyses.

### 3.2 Association Between the Environmental Variables and Macroinvertebrates

**FIGURE 4:**
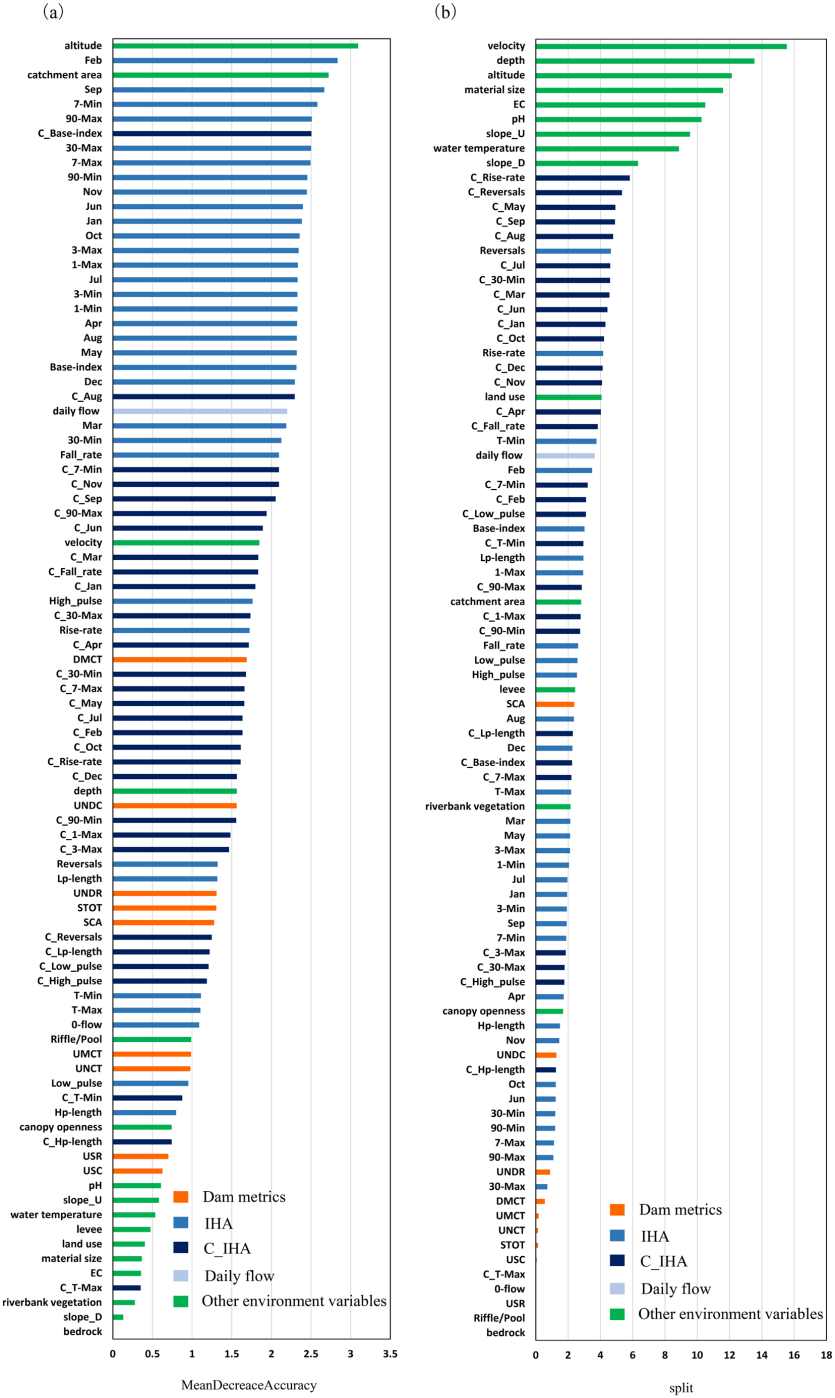
Variable importance generated based on the (a) random forest and (b) light gradient boosting machine (LightGBM) algorithms.

Variable importance analyses based on RF and LightGBM revealed that the contributing factors determining the distribution of macroinvertebrate habitats were altitude, catchment area, hydraulics (e.g., velocity and depth), median discharge (e.g., February and September), low flow metrics (e.g., base index), and change ratio of median discharge (e.g., C_August and C_Sep) (Figure 4). Among the dam metrics, the UNDC and the catchment area of the river section fragmented by dams (SCA) showed greater importance than that of the other metrics.

Although IHA was relatively more important in the RF algorithm, C_IHA tended to be more important in LightGBM, probably owing to the differences in the importance calculation methods used. The IHA is inter‐correlated and has similar characteristics in certain metrics, whereas C_IHA is relatively less inter‐correlated. Therefore, with an importance analysis method “split,” which counts the number of times a variable is used in model building, the relatively fair importance values among the C_IHA were produced in the LightGBM algorithm, as a wide range of the C_IHA was used owing to their different characteristics.

### 3.3 Contributions of the Dam Metrics and IHA to Macroinvertebrate Distributions

**FIGURE 5:**
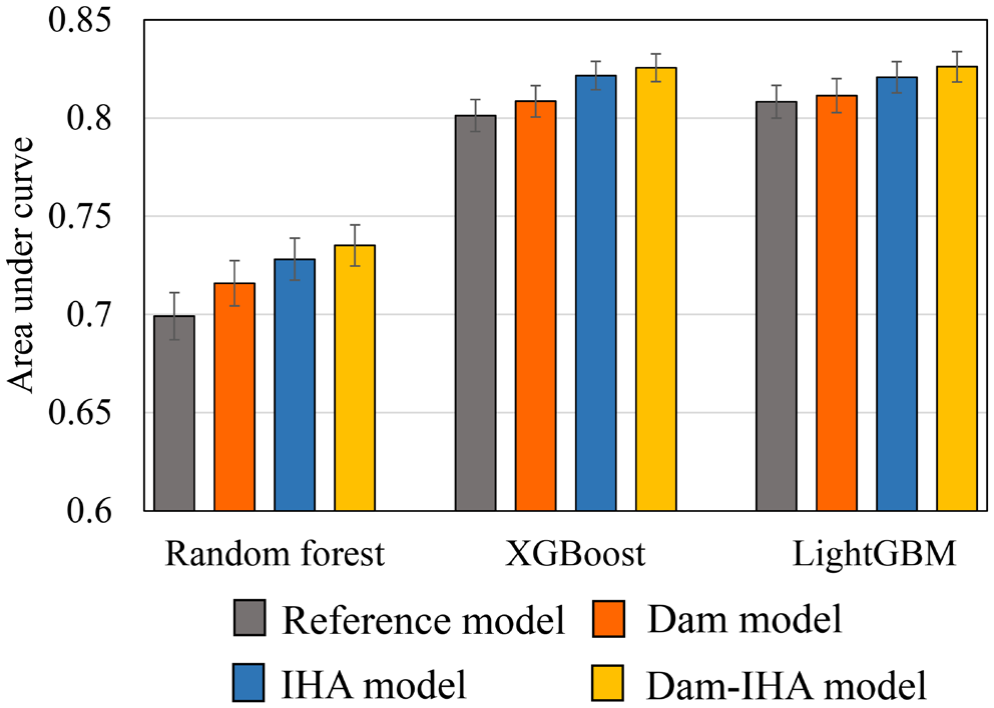
Mean area under curve (AUC) among the models of 170 macroinvertebrate taxa with selected predictors for the random forest, extreme gradient boosting (XGBoost), and light gradient boosting machine (LightGBM) algorithms. Here, the “reference model” refers to a model that excludes the dam index/indicators of hydrologic alteration (IHA) and half of the other variables with low importance; the “dam model” refers to a model that includes the dam metrics in the reference model; the “IHA model” refers to a model that includes the IHA in the reference model; and the “dam–IHA model” refers to a model that includes both the dam metrics and IHA in the reference model.

The mean AUC among the 170 taxa of macroinvertebrates for all models, except for the reference model of the RF, satisfied the criterion of good model accuracy of 0.7 (Figure 5) (Swets 2015). The dam–IHA model for LightGBM yielded the highest mean accuracy with an average AUC of 0.826. All machine learning algorithms improved the average AUC in the models with dam metrics and IHA, suggesting the usefulness of these metrics in predicting the distribution of the macroinvertebrate community.

**FIGURE 6:**
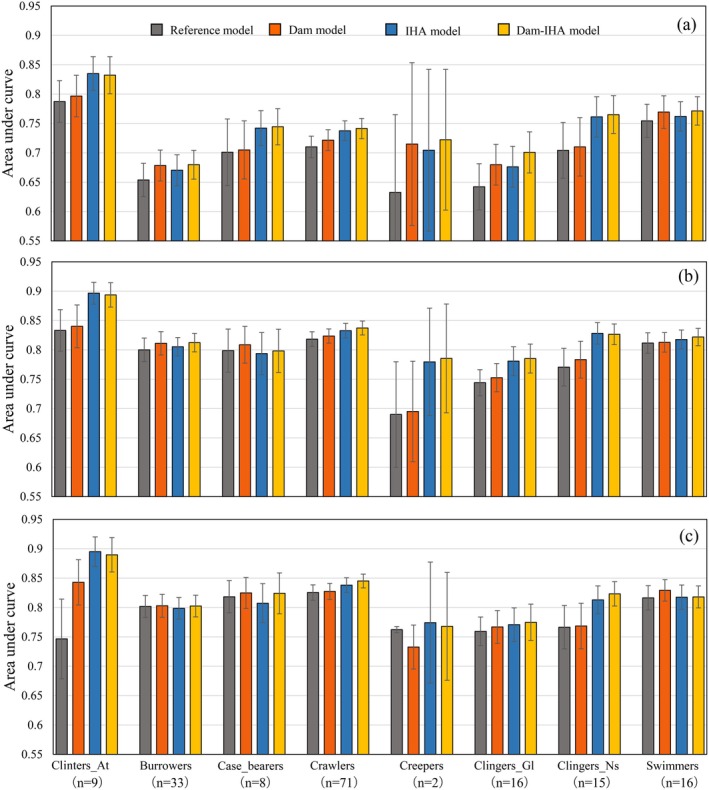
Mean area under curve (AUC) in the models of eight habit groups integrating the selected predictors for the random forest (a), extreme gradient boosting (XGBoost) (b), and light gradient boosting machine (LightGBM) (c) algorithms. Details of the four models (including the reference model) are described in the main text and the caption for Figure 4.

Figure 6 shows the mean AUC among the taxa of the eight habit groups for all models. The reference models for the RF, XGB, and LightGBM algorithms attained AUC > 0.7 for five, seven, and eight groups, respectively. The accuracies for each algorithm were greatly improved with the dam metrics and IHA, and the models with the dam metrics and IHA for RF, XGB, and LightGBM attained the criteria for the 6–7, 7–8, and 8 groups, respectively. Based on these results, the variable importance was evaluated in modeling the habit groups for which the AUC clearly increased. The other groups with unchanged AUC were not further analyzed because these results imply that the dam metrics are less important or redundant for these groups.

**FIGURE 7:**
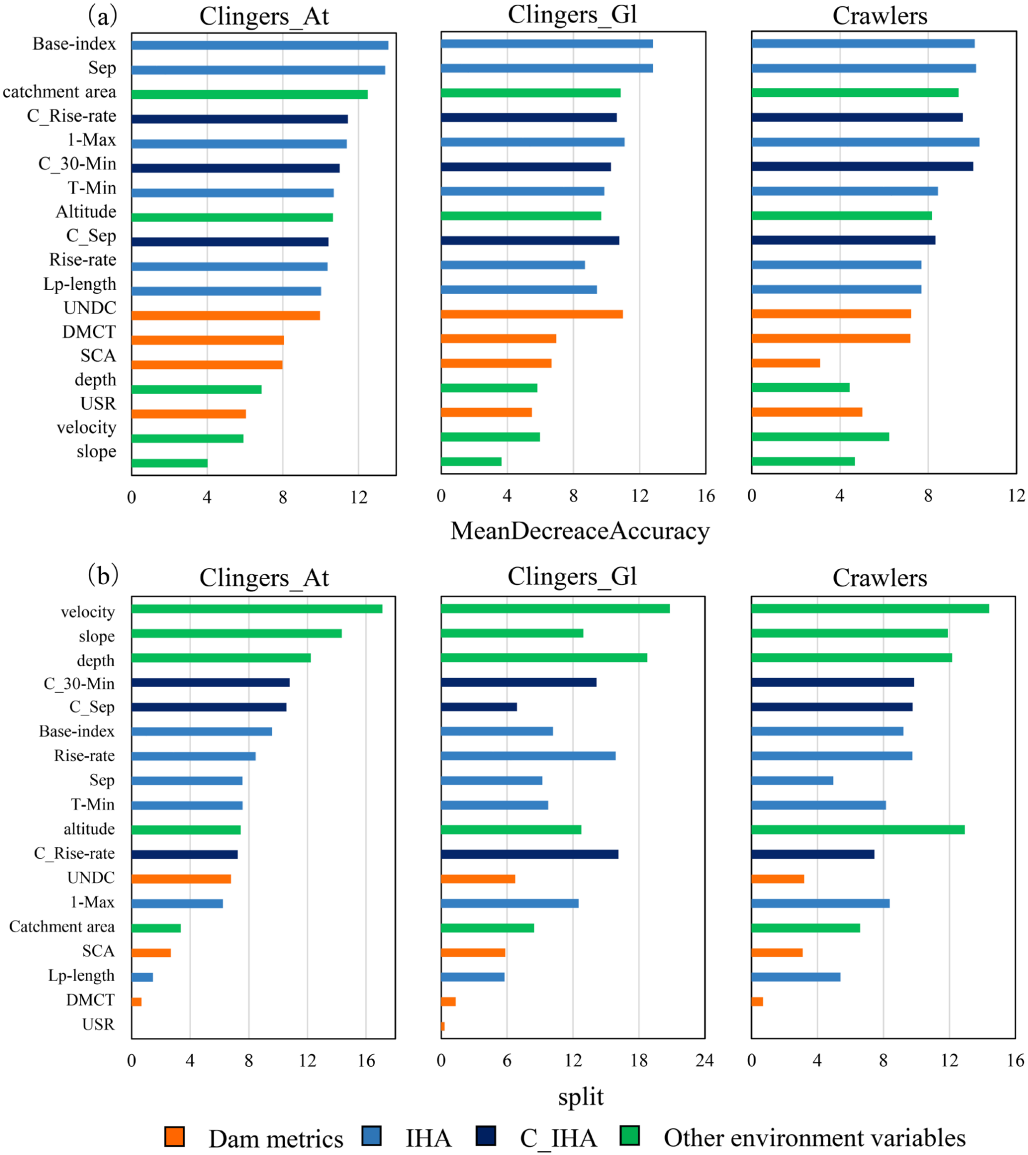
Mean variable importance among the models of three habit groups for the random forest (a) and light gradient boosting machine (LightGBM) (b) algorithms. Here, the range of negative importance values is excluded for visibility reasons.

The accuracy tended to increase with IHA in most algorithms for Clingers_At, a variant of clingers with a sessile habit with/without a case, such as Simuliidae and *Limnocentropus* sp., and Clingers_Gl, a group that includes Heptageniidae and Psephenidae sp. (Figure 6). The important variables of IHA included extremely high‐flow metrics (e.g., median flow in September) and low‐flow metrics (e.g., base‐flow index) for these habit groups (Figure 7).

## 4 Discussion

In the present study, we successfully improved the habitat models of macroinvertebrate community by incorporating dam impact metrics (i.e., dam metrics and IHA) and sophisticated GBMs with optimized hyperparameters, such as ESR. We found that a parameter that controls the number of trees is key to increasing the average prediction accuracy among hundreds of species in a specific community. In addition, the average accuracies were much higher than those based on RF, which repeatedly yield better performances than those of popularly used algorithms (Hao et al. 2020). Therefore, we recommend using either boosting machine techniques while adjusting the critical hyperparameters when modeling several species, such as studying community ecology (e.g., joint species distribution models) (Zurell et al. 2020).

We found higher importance of IHA than that of the dam metrics, suggesting that, among the impacts of dams, alteration of the flow regime could be a major influential factor determining the distribution of macroinvertebrates. Specifically, high‐flow and base‐flow metrics (September median, 1‐day maximum, and base‐index) were relatively important for the studied habit groups. Flow regimes are critical metrics for the distribution of riverine species as these modify their habitable areas and life cycles (Lytle and Poff 2004). For example, the habitat of the endangered Gangetic dolphin has declined owing to the alteration in flow regime due to climate change (Sharma et al. 2022). Therefore, flow alteration could be a major effect among dam‐induced environmental changes. Notably, a previous study documented that flow regulation negatively affects ecosystems, regardless of the spatial configuration of dams (Grill et al. 2014). Hence, implementing dam metrics alone is insufficient; therefore, flow metrics must also be incorporated into habitat modeling for better applicability to other catchments or periods (i.e., transferability) (Hao et al. 2020). Recent studies have demonstrated that the transferability of species distribution models for stream macroinvertebrates is often limited under novel environmental conditions such as drought, with only slightly better performance observed for algorithms like spatial stream networks (SSN) and random forests (RF) (e.g., Medina‐Madariaga et al. 2025). These findings highlight that while our optimized models show improved performance within the study catchment, caution is required when extrapolating to other regions or under future climate extremes. In doing so, future studies should incorporate ecologically relevant predictors while considering extreme conditions.

Among the dam metrics, the number of dams per unit catchment area (UNDC) and the catchment area of the river section fragmented by dams (SCA) showed the highest importance. Although the prominent effect of dam density metrics (UNDC) is consistent with the results of previous studies targeting freshwater mussels and fish (Daniel et al. 2018; Mollenhauer et al. 2021), the relatively high contribution of such a fragmentation effect contradicts the results of a previous study (Grenouillet et al. 2008). These differences can presumably be ascribed to the following reasons: (1) the deterioration of the recovery function in fragmented catchments rather than due to fragmentation itself (see below), (2) the differences in identification level, e.g., family level (Grenouillet et al. 2008), and (3) the differences in dam characteristics, e.g., height (J. Wang et al. 2020). As the SCA represents the catchment area of fragmented segments, it probably reflects the potential for community recovery after flow disturbance through invertebrate immigration from tributaries (Gabbud et al. 2019). Furthermore, a previous study demonstrated that genetic structures between populations up‐ and downstream of dams for some macroinvertebrate species significantly differed, suggesting that damming interrupts migration along a watercourse (Watanabe et al. 2010). Thus, the results of the present study suggest that fragmentation affects the distribution of many macroinvertebrates by reducing their recovery potential.

The results of the variable importance analysis also highlighted the contributions of hydraulic variables, likely because our sampling design targeted a broad range of hydraulic conditions (samples from both riffle and pool). Hydraulics are critical habitat components for stream macroinvertebrates (Mérigoux and Dolédec 2004; Poff et al. 2006); however, studies on riverine habitat modeling or species distribution modeling have rarely incorporated such metrics, although studies using model‐based hydraulics and targeting fish have been conducted (Nukazawa et al. 2023; Suzuki et al. 2021). A likely explanation for the lack of such studies is that the sampling of macroinvertebrates is typically performed in flowing water, whereas such a sampling design would not cover species that favor slow water current. These findings imply potential usefulness in expanding the conventional assessment framework to those including pool or lentic water bodies.

The extreme high‐flow metrics, e.g., median flow in September, and low‐flow metrics such as base‐flow index, were observed to be critical variables in modeling the habit group of clingers. The flow in September reflects a typhoon‐induced high flow; therefore, the extent of shear stress during this season may constrain the settlement of these groups. Previous studies reported a decreased abundance of clingers under low‐flow conditions during drought seasons (Herbst et al. 2019) and anthropogenic perturbations in riverine communities (Liu et al. 2021; Maloney and Feminella 2006). In the present study, we also observed a keen response to low‐flow metrics, even in catchments with high annual rainfall, presumably owing to the alteration in flow caused by the dams; the downstream sections of dams receive only residual flow, except during the flooding period (Mineda et al. 2023). Notably, the change ratio for the minimum flow (C_30‐Min) showed greater importance than the other metrics. In addition, such a manipulation suppressed the rise rate for two consecutive days, which showed higher importance for Clingers_Gl than the other metrics. A previous study in an adjacent catchment reported that the abundance of taxa belonging to Clingers_Gl significantly decreased downstream of the dams because of habitat degradation associated with algal growth (Nukazawa, Kajiwara, et al. 2020). Hence, the suppressed flow promoted algal growth and constrained the inhabitation of the group.

Upon incorporating the dam metrics and IHA, the accuracies for Crawlers and Clingers_Gl tended to increase. These results suggest that many taxa in these habit groups determine their distributions in response to dam‐induced alterations, including flow regime alterations. The 1‐day maximum flow rate tended to be important for crawlers. Crawlers such as the Ephemerellidae sp. crawl on the surfaces of large substrate materials. This finding implies that riverbed disturbances caused by extremely high flows, which promote passive drifts, also regenerate material structures and affect their distribution.

## 5 Conclusions

In the present study, we demonstrated that the metrics of dam‐induced environmental impacts are useful for the habitat modeling of stream macroinvertebrate communities at a catchment scale. We found that flow metrics that include dam impacts (e.g., abstraction) were more important than simple metrics of dam impacts based on geographical information systems. We compared cutting‐edge ensemble learning techniques (RF, XGB, and LightGBM) and found that the gradient‐boosting methods with a parameter optimizing tree number for each taxon are promising for predicting the distributions of the biotic community. In another aspect, samples from a broader range of hydraulic conditions (e.g., riffle and pool) were suggested to improve habitat modeling of macroinvertebrates.

The habitat models developed are likely to be more applicable to other catchments or periods (i.e., transferable) (Hao et al. 2020) because numerous small to large catchments were altered by dams and weirs in the Anthropocene. To test this hypothesis, future studies should transfer our models to other catchments or periods to evaluate temporal and spatial transferability. Given that such a challenge is successfully addressed, models will be helpful when river practitioners implement riverine conservation measures as they better understand the environmental consequences of their flood protection designs with damming or considerable environmental changes such as flow regime alteration.

## Author Contributions

**Kei Nukazawa:** conceptualization (lead), funding acquisition (lead), investigation (lead), methodology (lead), supervision (lead), validation (equal), writing – original draft (equal), writing – review and editing (lead). **Ryo Tanaka:** data curation (equal), formal analysis (lead), investigation (supporting), methodology (supporting), validation (equal), writing – original draft (equal). **Haruki Mineda:** data curation (equal), formal analysis (supporting), writing – review and editing (supporting).

## Conflicts of Interest

The authors declare no conflicts of interest.

## Supporting information


**Appendix S1:** ece372411‐sup‐0001‐AppendixS1.docx.

## Data Availability

Macroinvertebrate and environmental data are available at the University of Miyazaki Academic Repository; https://doi.org/10.34481/0002001366.
