# Supplementary material for: Importance of Flow Metrics on Modeling Macroinvertebrate Community in Dammed Rivers: An Approach With Optimized Gradient Boosting
Source: Ecol Evol. 2025 Oct 28;15(10):e72411. doi: 10.1002/ece3.72411 (PMC12559814; doi:10.1002/ece3.72411)
Supplement: Supplementary file 1 — Appendix S1: ece372411‐sup‐0001‐AppendixS1.docx. [file ECE3-15-e72411-s001.docx]

**Importance of flow metrics on modeling macroinvertebrate community in dammed rivers: an approach with optimized gradient boosting**

Nukazawa K.*, Tanaka R., Mineda H.

*Kei Nukazawa (Corresponding author)

Department of Civil and Environmental Engineering, Faculty of Engineering, University of Miyazaki, Gakuen Kibanadai-nishi 1-1, Miyazaki 889-2192, Japan

e-mail: nukazawa.kei.b3@cc.miyazaki-u.ac.jp

## Supplemental Figures and Tables

## Supplemental Figures


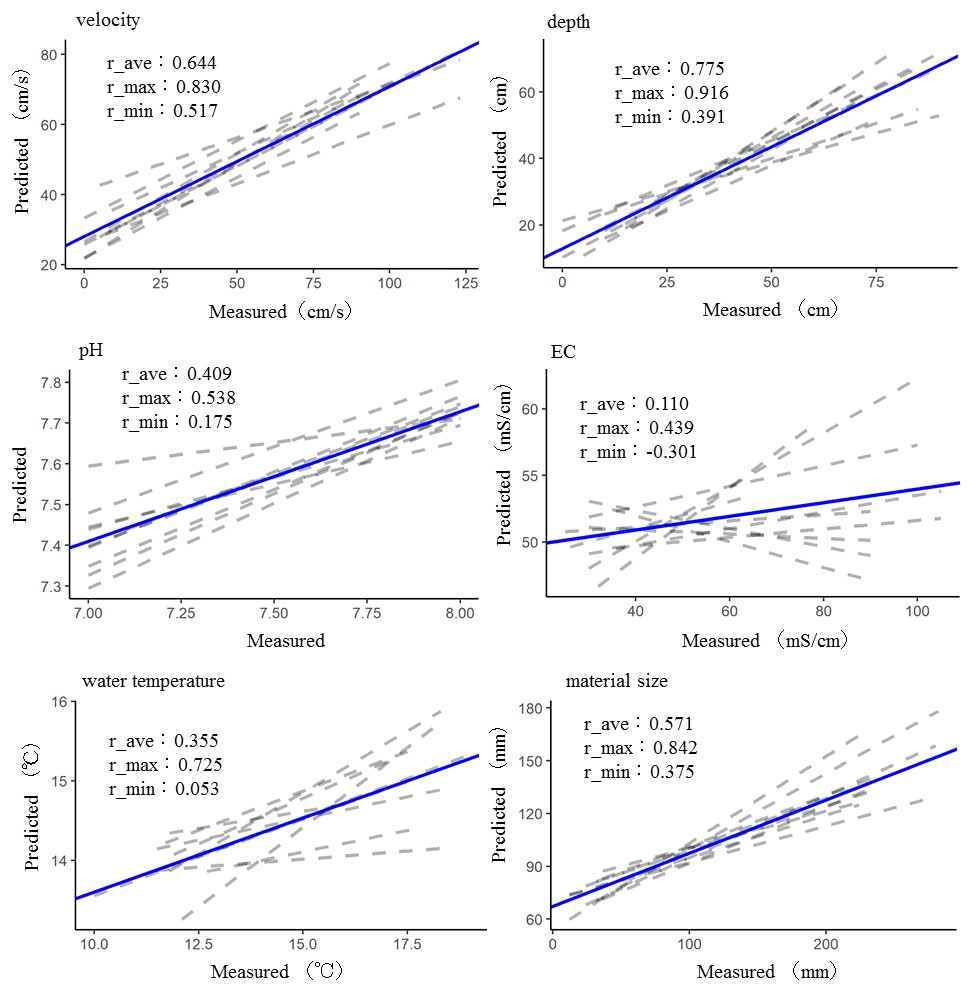


**Figure S1** Results of interpolations of environmental variables; blue line indicates prediction by linear mixed model (blue line) with fix effect of predicted parameters and random effect of each split of cross-validation while broken ash lines indicate linear models for each split


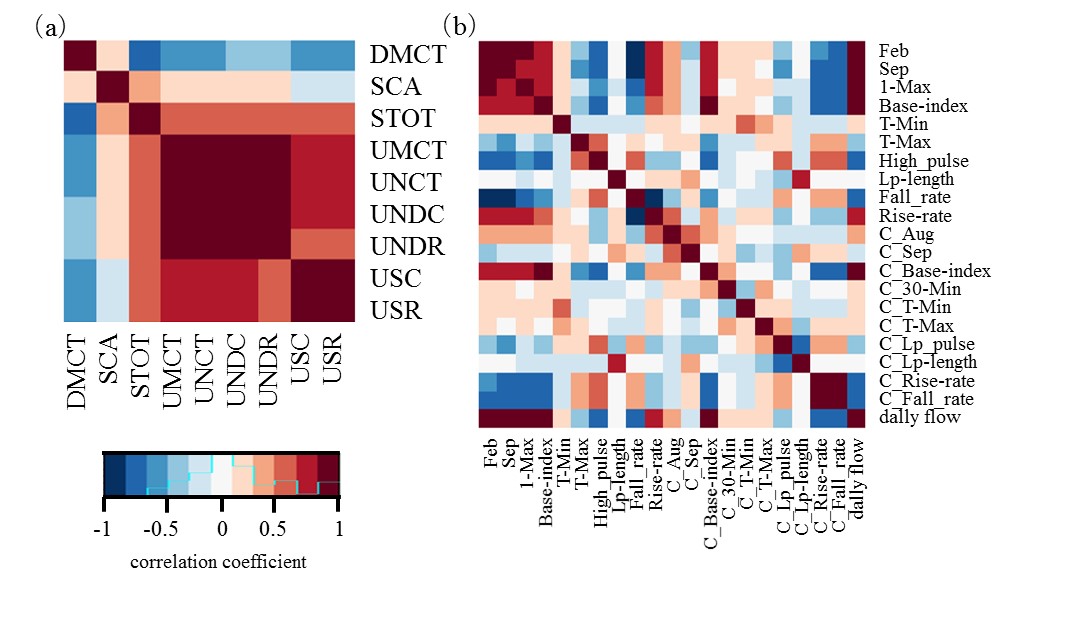


**Figure S2** Spearman’s rank correlation among dam metrics (a) and IHA (b). Note that the two most important IHA based on random forest and LightGBM are used.


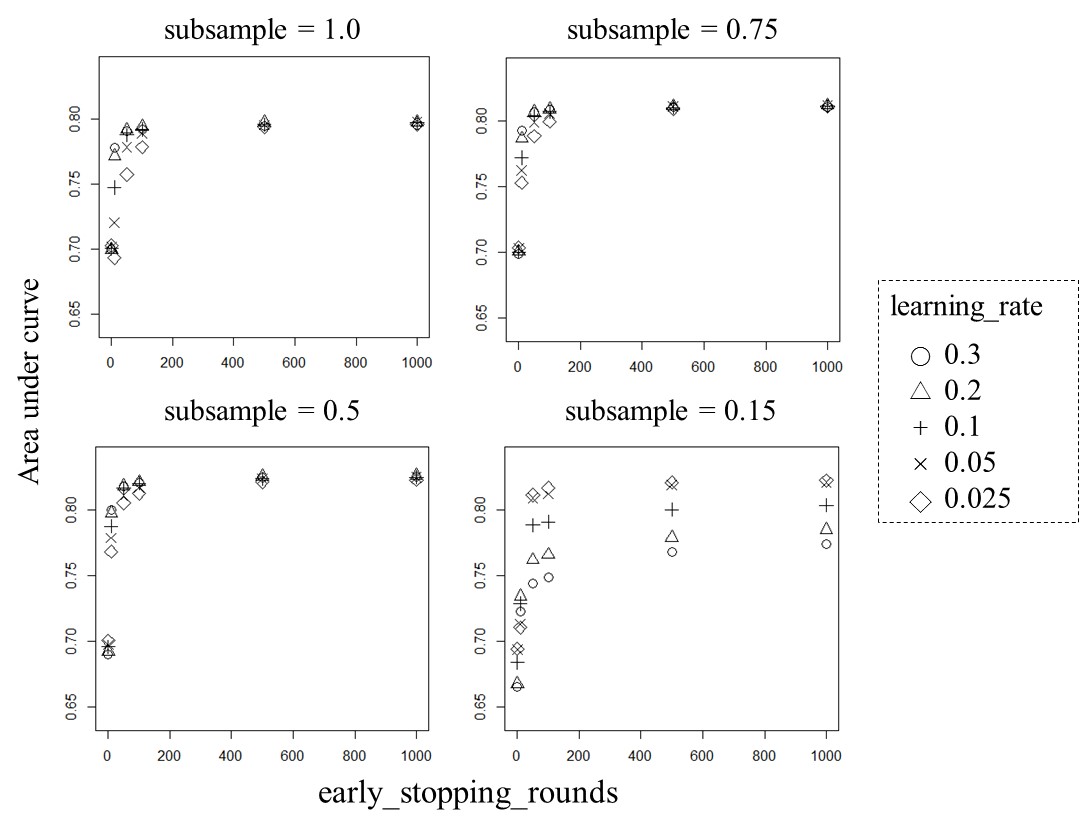


**Figure S3** Sensitivity analysis for XGBoost. Area under curve was mean of 170 macroinvertebrate taxa.


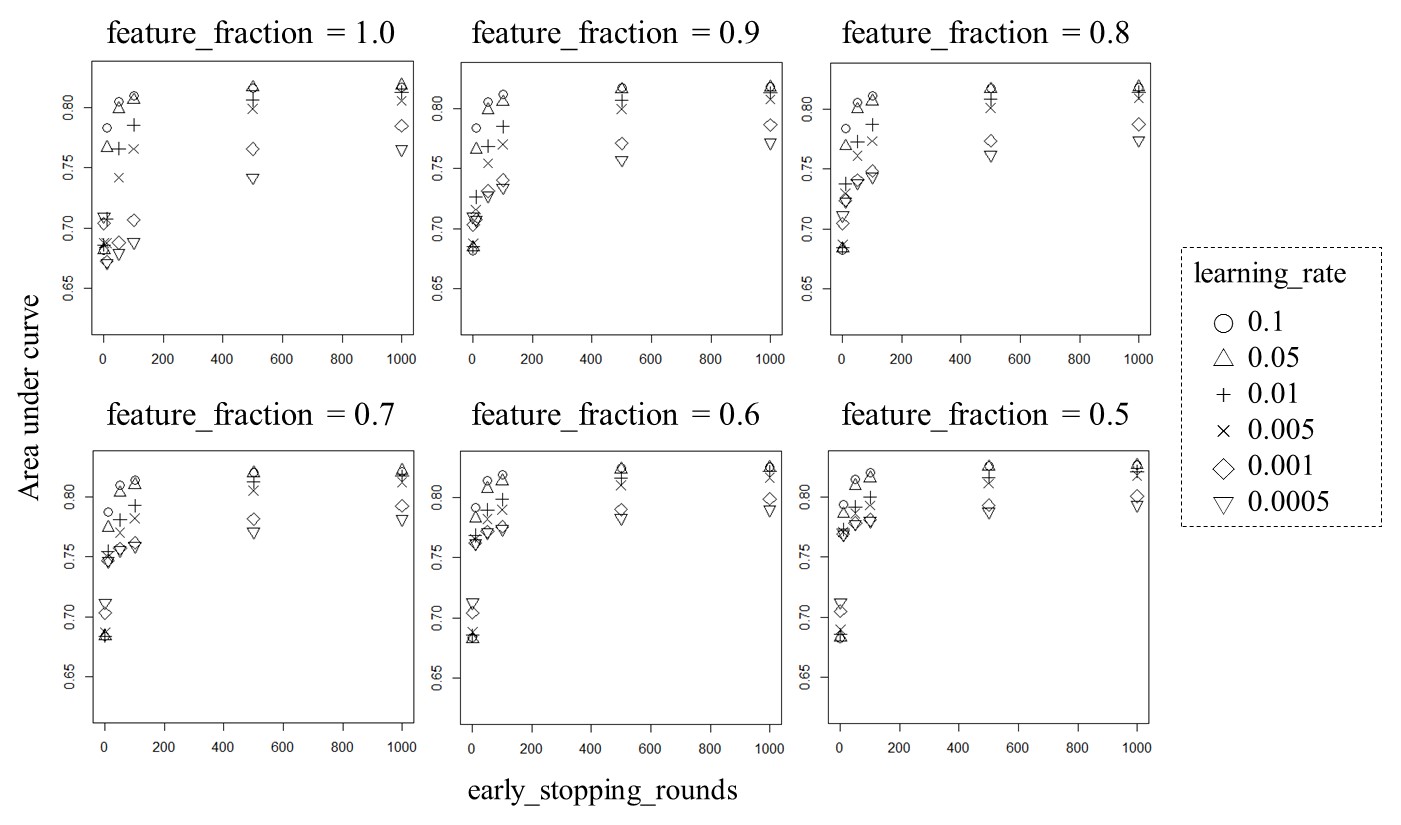


**Figure S4** Sensitivity analysis for LightGBM. Area under curve was mean of 170 macroinvertebrate taxa.

**Supplemental Tables**

**Table S1** Presence or absence of 171 macroinvertebrate taxa modeled in the study (available at the University of Miyazaki Academic Repository; https://doi.org/10.34481/0002001366).

**Table S2** Environmental parameters used in the study (available at the University of Miyazaki Academic Repository; https://doi.org/10.34481/0002001366).

**Table S3** Habit groups in the present study and their correspondence to the previous studies

| Habit group | |  | Classification in  Merritt and Cummins (1996) |
| --- | --- | --- | --- |
| This study | Takemon, 2005 | Representative |  |
| Clingers_At | Attachers | *Simulium* sp., Simuliidae, *Agathon* sp. | Clingers |
| Burrowers | Burrowers | *Orthocladius* sp., *Polypedilum* sp., *Choroterpes altioculus* | Burrowers, Sprawlers |
| Case-bearers | Case-bearers | *Lepidostoma* sp., *Glossosoma* sp., *Ceraclea* sp. | Sprawlers, Climbers |
| Crawlers | Crawlers | *Cincticostella elongatula*, *Chloroperlidae*, *Neoperla* sp. | Sprawlers, Climbers, Clingers |
| Clingers_Gl | Gliders | *Rhithrogena* sp., *Ecdyonurus* sp., *Epeorus latifolium* | Clingers |
| Creepers | Creepers | *Dugesia japonica*, *Semisulcospira reiniana* | Sprawlers, Clingers, Climbers |
| Clingers_Ns | Net-spinners | *Hydropsyche* sp., *Cheumatopsyche infascia*, *Cheumatopsyche* sp. | Clingers |
| Swimmers | Swimmers | *Baetiella japonica*, *Baetis taiwanensis*, *Nigrobaetis* sp. D | Swimmers, Divers, Climbers |

**Table S4** Environmental predictor variables, the number of site where the metrics were obtained, and basic statistics (i.e., average and range) among the study sites. Note that the statistics of categorical variables are not shown here.

| Metric | Study site | Average | Range |
| --- | --- | --- | --- |
| Riverbank vegetation | 83 | - | - |
| Canopy openness | 83 | - | - |
| Levee | 83 | - | - |
| Bedrock | 83 | - | - |
| Riffle/Pool | 83 | - | - |
| Altitude | 83 | 222.73 | 1.73~792.52 |
| Catchmant area | 83 | 116.29 | 2.75~441.19 |
| Landuse | 83 | - | - |
| Slope_U | 83 | 4.93 | 5.00×10^-3^~34.571 |
| Slope_D | 83 | 0.21 | 0.00~10.55 |
| Flow velocity | 83 | 47.23 | 4.14~123.45 |
| Water depth | 83 | 31.68 | 8.38~76.42 |
| Water temperature | 83 | 14.86 | 11.50~18.00 |
| pH | 83 | 7.61 | 7.12~8.47 |
| EC | 83 | 50.21 | 26.20~102.80 |
| Riverbed material size | 83 | 95.65 | 12.40~280.66 |

**Table S5** Dam metrics proposed in the previous study (Cooper et al., 2017), the number of site where the metrics were calculated, and basic statistics (i.e., average and range) among the study sites.

| Metric | Description | Study site | Average | Range |
| --- | --- | --- | --- | --- |
| Segment-based | |  |  |  |
| STOT | Total segment length(km) | 83 | 128.32 | 19.81~162.20 |
| SMST | Segment mainstem length(km) | 46 | 21.42 | 4.42~24.15 |
| SCA^a^ | Segment catchment area(km^2^) | 83 | 110.41 | 24.00~142.81 |
| Count and density | |  |  |  |
| UNCT | Total upstream dam count(#) | 83 | 1.23 | 0.00~6.00 |
| UMCT | Upstream mainstem dam count(#) | 83 | 1.05 | 0.00~5.00 |
| DMCT^a^ | Downstream mainstem dam count(#) | 83 | 3.14 | 0.00~5.00 |
| UNDR | Upstream network dam density per unit stream network length(#/km^2^) | 83 | 6.61×10^-3^ | 0.00~2.05×10^-2^ |
| UNDC^a^ | Upstream network dam density per unit network catchment area(#/km) | 83 | 5.48×10^-3^ | 0.00~1.68×10^-2^ |
| UMD^b^ | Upstream mainstem dam density per unit upstream mainstem length(#/km) | 46 | 4.40×10^-2^ | 0.00~8.68×10^-2^ |
| DMD^b^ | Downstream mainstem dam density per unit downstream mainstem length(#/km) | 46 | 5.39×10^-2^ | 0.00~9.08×10^-2^ |
| Distance-based | |  |  |  |
| UM2D^b^ | Distance to upstream mainstem dam(km) | 38 | 11.25 | 0.42~19.50 |
| DM2D^b^ | Distance to downstream mainstem dam(km) | 64 | 14.39 | 0.25~29.47 |
| UMO^b^ | Percentage of open upstream mainstem(%) | 46 | 44.98 | 2.52~100.00 |
| DMO^b^ | Percentage of open downstream mainstem(%) | 46 | 45.45 | 0.37~100.00 |
| Cumulative reservoir storage | |  |  |  |
| USR^a^ | Upstream reservoir storage volume per unit stream network length(km^3^/km) | 83 | 4.13×10^-5^ | 0.00~3.90×10^-4^ |
| USC | Upstream reservoir storage volume per unit stream network catchment area(km^3^/km) | 83 | 3.73×10^-5^ | 0.00~3.82×10^-4^ |
| ^a^ Metrics used in dam models | |  |  |  |
| ^b^  Metrics excluded from the habitat model due to the small number of sites included in the calculation | |  |  |  |

**Table S6** Detailed information of indicators of hydrologic alteration (Richter et al., 1996), sample size, and basic statistics (i.e., average and range) among the study sites.

| Metrics | Description | Sample size | Average | Range |
| --- | --- | --- | --- | --- |
| Magnitude of monthly water conditions | |  |  |  |
| Jan | Median flow in January(m^3^/s) | 107 | 1.21 | 1.90×10^-2^~5.34 |
| Feb | Median flow in February(m^3^/s) | 107 | 2.07 | 3.90×10^-2^~11.87 |
| Mar | Median flow in March(m^3^/s) | 107 | 5.52 | 0.17~27.51 |
| Apr | Median flow in April(m^3^/s) | 107 | 2.33 | 7.30×10^-2^~9.91 |
| May | Median flow in May(m^3^/s) | 107 | 6.17 | 0.10~28.32 |
| Jun | Median flow in June(m^3^/s) | 107 | 9.89 | 0.23~36.73 |
| Jul | Median flow in July(m^3^/s) | 107 | 17.24 | 0.34~60.95 |
| Aug | Median flow in August(m^3^/s) | 107 | 10.75 | 8.20×10^-2^~51.20 |
| Sep | Median flow in September(m^3^/s) | 107 | 12.96 | 0.17~60.96 |
| Oct | Median flow in October(m^3^/s) | 107 | 5.76 | 5.40×10^-2^~38.89 |
| Nov | Median flow in November(m^3^/s) | 107 | 2.13 | 4.40×10^-2^~25.64 |
| Dec | Median flow in December(m^3^/s) | 107 | 2.33 | 2.00×10^-3^~20.32 |
| Magnitude and duration of annual extreme water conditions | |  |  |  |
| 1-Min | 1-day minimum flow rate(m^3^/s) | 107 | 0.70 | 0.00~3.88 |
| 3-Min | 3-day minimum flow rate(m^3^/s) | 107 | 0.86 | 0.00~4.08 |
| 7-Min | 7-day minimum flow rate(m^3^/s) | 107 | 0.93 | 1.43×10^-4^~4.40 |
| 30-Min | 30-day minimum flow rate(m^3^/s) | 107 | 1.26 | 1.04×10^-2^~7.37 |
| 90-Min | 90-day minimum flow rate(m^3^/s) | 107 | 3.17 | 7.34×10^-2^~13.18 |
| 1-Max | 1-day maximum flow rate(m^3^/s) | 107 | 251.22 | 5.48~1059.50 |
| 3-Max | 3-day maximum flow rate(m^3^/s) | 107 | 155.25 | 3.70~628.34 |
| 7-Max | 7-day maximum flow rate(m^3^/s) | 107 | 100.47 | 1.96~379.38 |
| 30-Max | 30-day maximum flow rate(m^3^/s) | 107 | 44.61 | 1.08~162.86 |
| 90-Max | 90-day maximum flow rate(m^3^/s) | 107 | 29.51 | 0.68~103.03 |
| 0-flow | Number of days for flow rate 0(days) | 107 | 0.86 | 0.00~15.00 |
| Base-index | base current index(m^3^/s) | 107 | 0.04 | 4.02×10^-4^~0.14 |
| Timing of annual extreme water conditions | |  |  |  |
| T-Min | Date of minimum flow occurrence(day) | 107 | 190.83 | 3.00~361.00 |
| T-Max | Date of maximum flow occurrence(day) | 107 | 252.92 | 180.00~273.00 |
| Frequency and duration of high and low pulses | |  |  |  |
| Low_pulse | Low pulse generation frequency(Number) | 107 | 16.08 | 7.00~27.00 |
| Lp-length | Low pulse duration days(days) | 107 | 3.70 | 1.00~6.00 |
| High_pulse | High pulse generation frequency(Number) | 107 | 16.07 | 9.00~31.00 |
| Hp-length | High pulse duration days(days) | 107 | 3.91 | 1.00~7.00 |
| Rate and frequency of water condition changes | |  |  |  |
| Rise-rate | Rising values for two consecutive days(m^3^/s) | 107 | 1.08 | 8.00×10^-3^~3.69 |
| Fall_rate | Descending value for 2 consecutive days(m^3^/s) | 107 | -0.62 | -2.62~-3.0×10^-3^ |
| Reversals | Number of reversals of increase/decrease in flow over two consecutive days(Number) | 107 | 135.57 | 120.00~169.00 |

**Table S7** Parameters considered to be tuned in the present study as well as identified values for parameter used in the final models

| Parameter | | Description | Identified values |
| --- | --- | --- | --- |
| XGBoost | |  |  |
|  | colsample_bylevel | The subsample ratio of columns for each level. |  |
|  | early_stopping_round | Model will stop training if one metric of one validation data doesn’t improve in last early_stopping_round rounds | 500 |
|  | gamma | Minimum loss reduction required to make a further partition on a leaf node of the tree. |  |
|  | learning_rate | Step size shrinkage used in update to prevents overfitting. | 0.1 |
|  | max_depth | Maximum depth of a tree. |  |
|  | num_boost_round | The number of boosting iterations. | 10000 |
|  | subsample | Subsample ratio of the training instances. | 0.5 |
| LightGBM | |  |  |
|  | bagging_fraction | Rows are sampled randomly from the training data and the percentage used in each iteration is specified. |  |
|  | early_stopping_round | Model will stop training if one metric of one validation data doesn’t improve in last early_stopping_round rounds | 500 |
|  | feature_fraction | A subset of features on each iteration (tree) is randomly selected if feature_fraction is smaller than 1.0. | 0.5 |
|  | learning_rate | Step size shrinkage used in update to prevents overfitting. | 0.1 |
|  | max_depth | Maximum depth of a tree. |  |
|  | min_data_in_leaf | Minimal number of data in one leaf. |  |
|  | min_sum_hessian_in_leaf | Minimal sum hessian in one leaf. |  |
|  | num_boost_round | Specifies the number of boosting iterations. | 10000 |
|  | num_leaves | Max number of leaves in one tree |  |
